# Retrospective Analysis of a Seborrheic Keratosis–Like Melanoma on the Head

**DOI:** 10.7759/cureus.52678

**Published:** 2024-01-21

**Authors:** Jesús Iván Martínez-Ortega, Arely Ramirez Cibrian

**Affiliations:** 1 Department of Dermatology, Dermatological Institute “Dr. José Barba Rubio”, Zapopan, MEX; 2 Department of Medical Affairs, Mexican Institute of Social Security, Campeche, MEX

**Keywords:** clinical case, diagnostic challenges, seborrheic keratosis, melanoma, dermoscopy

## Abstract

We present a clinical case of a 50-year-old female initially suspected of seborrheic keratosis but later diagnosed with melanoma through biopsy. This case highlights the challenges in distinguishing between these two conditions and emphasizes the importance of accurate diagnosis. Overdiagnosis of malignancy in seborrheic keratosis cases and the accurate identification of melanoma through dermoscopy are discussed. Further research is needed to explore potential mechanistic connections between seborrheic keratosis and melanoma.

## Introduction

Seborrheic keratoses (SKs), which are benign epithelial tumors, are commonly encountered in clinical practice. Despite their benign nature, they are often biopsied and removed for cosmetic reasons. These growths tend to appear in areas with hair, such as the head, neck, trunk, and extremities. SKs are generally asymptomatic and typically manifest with a distinctive and easily identifiable clinical aspect of clearly defined verrucous or wax-like cutaneous growths, characterized by a superficial stuck-on appearance and keratotic plugs on their surface [1-3]. In the context of these benign lesions, we explore a case involving a 50-year-old female without known comorbidities. She presented with a symptom-free lesion on the crown of her head for six months. While initially suspected to be SK due to its appearance, a biopsy revealed a surprising diagnosis of melanoma. Our analysis delves into the clinical, histopathological, and dermatoscopic features that played pivotal roles in this case. Furthermore, we delve into broader discussions surrounding the diagnostic challenges posed by the convergence of SK and melanoma characteristics, emphasizing the importance of accurate diagnosis and patient safety.

## Case presentation

**Figure 1:**
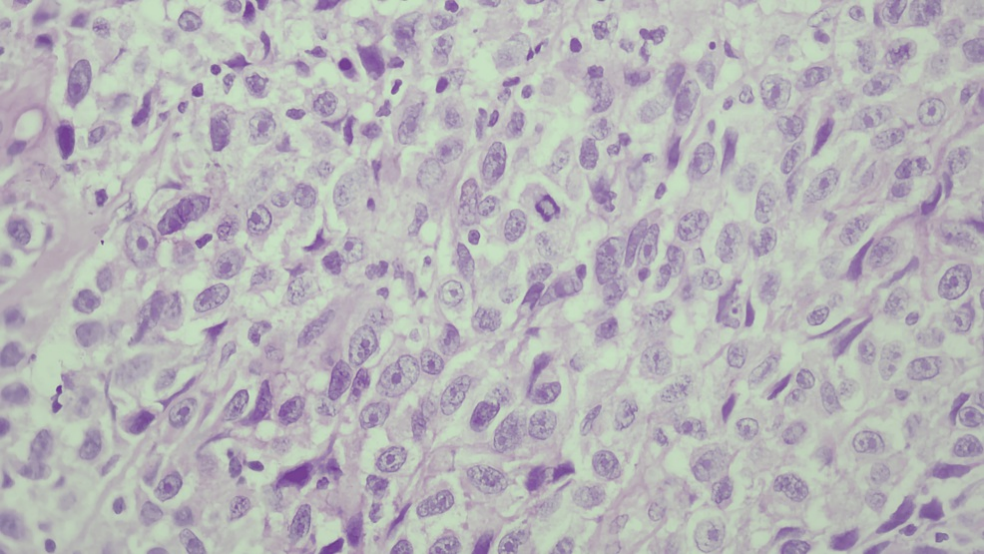
Histopathology Microscopic examination, H&E stain, at 40x magnification, reveals numerous atypical melanocytes of epithelioid appearance characterized by a pleomorphic nucleus and prominent nucleolus. H&E, hematoxylin and eosin

In the clinical case, a 50-year-old female with no known comorbidities presented with a symptom-free lesion on the crown of her head for six months. Seeking an initial consultation with a dermatologist SK was suspected due to the lesion's appearance. A biopsy was conducted, leading the patient to seek our evaluation upon discovering melanoma in the histopathology results. The received specimen, an incisional biopsy of hyperpigmented cutaneous neoformation on the scalp measuring 6x3x3 mm, was placed in formalin and embedded in paraffin for histopathological analysis. Microscopically, sections revealed numerous atypical melanocytes forming nests of varying sizes, showcasing an epithelioid appearance with a pleomorphic nucleus, and prominent nucleolus (Figure 1).

**Figure 2:**
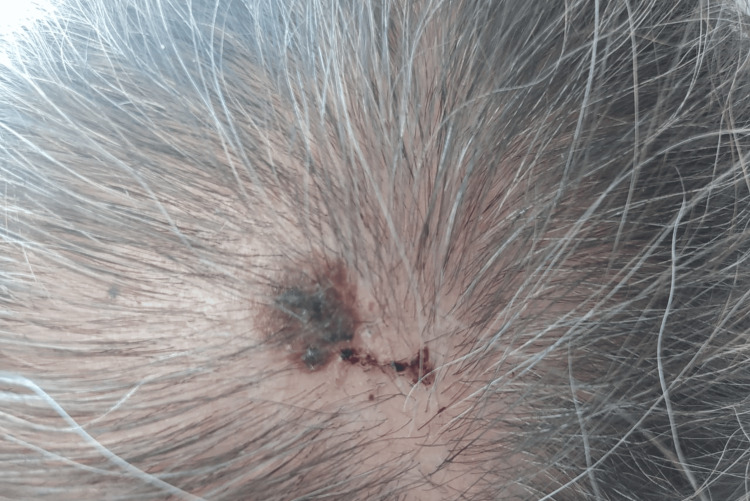
Clinical aspect of the lesion. Irregular seborrheic-like lesion situated on the crown of the patient's head.

Notably, no areas of regression, perineural infiltration, or lymphovascular invasion were observed. The interpretation indicated a superficially spreading malignant melanoma with vertical growth, an invasion level of Clark III, and a Breslow thickness of 1.4 mm. Regrettably, further patient follow-up was hindered as she opted for medical care in another country. Clinical examination revealed an irregular seborrheic-like lesion on the head's crown, with dermoscopy unveiling characteristic features including a pigment network at the lesion's periphery (indicated by a black arrowhead), suggesting a specific pattern (Figure 2).

**Figure 3:**
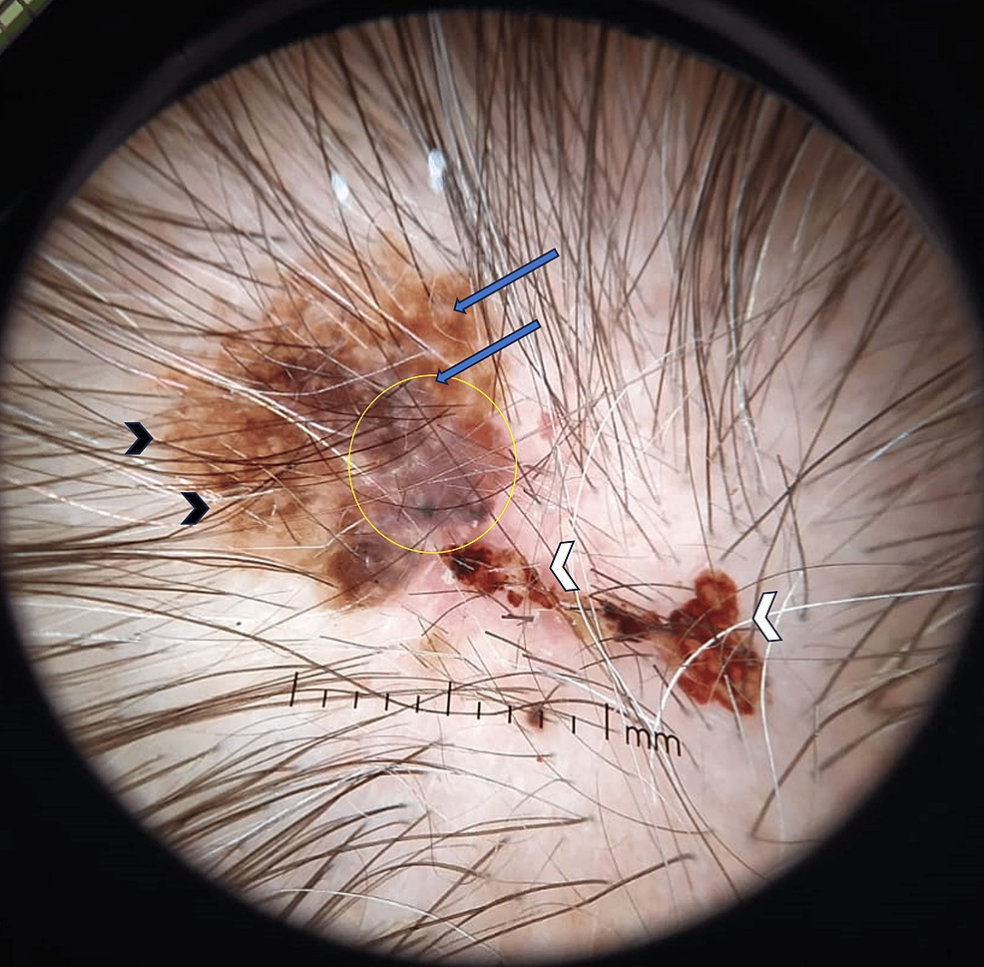
Dermoscopy image illustrating characteristic features of the observed skin lesion. A pigment network is evident at the periphery (black arrowhead). In the center, a distinct blue-white veil is observed (circle). Additionally, scattered milia-like cysts are visible within the lesion (blue arrow). The presence of hematic crusts is also noted (white arrowhead).

Additionally, there was a hematic crust on the lesion's surface (white arrowhead). At the lesion's center, a distinctive blue-white veil (circled) created striking contrast, and scattered milia-like cysts (blue arrow) contributed to its dermoscopic appearance (Figure 3).

The presence of hematic crusts on the lesion's surface (white arrowhead) further enriched the clinical presentation.

## Discussion

A comprehensive analysis of skin biopsy results from 8,694 patients revealed that 30% of excised SKs were inaccurately diagnosed as nonmelanocytic skin cancer, while 3% were misdiagnosed as melanoma. Intriguingly, among the misidentified cases of cutaneous melanoma, 21% were ultimately identified as SKs or benign nevi. These data underscore a tendency for malignancy to be overdiagnosed in such cases [4].

On the other hand, in an observational study by Carrera et al., involving 134 cases of melanoma, 82% of clinically SK-like melanomas were accurately identified through dermoscopy. Among the various criteria, the following signs were found to be most helpful in correctly diagnosing SK-like melanomas, listed in descending order of significance: pigmented network (55.2%), blue-white veil (53.7%), globules and dots (50.7%), pseudopods or streaks (35.1%), blue-black sign (32.3%), and milia-like cysts (22.4%). In the case discussed above, the presence of a pigment network and a blue-white veil were instrumental in raising suspicion for the diagnosis [5]. Despite the patient already having a histopathological diagnosis at the time of their visit to our clinic, the clinical impression and dermatoscopy findings mentioned in the pathology report revealed that the initial private dermatologist had already harbored suspicions based on the clinical and dermoscopic appearance. This proactive approach ultimately facilitated a prompt diagnosis of melanoma.

While there is a tendency to overdiagnose melanoma in cases with SK-like features, the more significant risk of overlooking SK-like melanomas compels us to address crucial aspects. In the absence of more refined and accurate diagnostic tools and biomarkers, it is prudent to prioritize patient safety. Therefore, in uncertain cases, a swift biopsy should be conducted to avert potential life-threatening outcomes.

**Figure 4:**
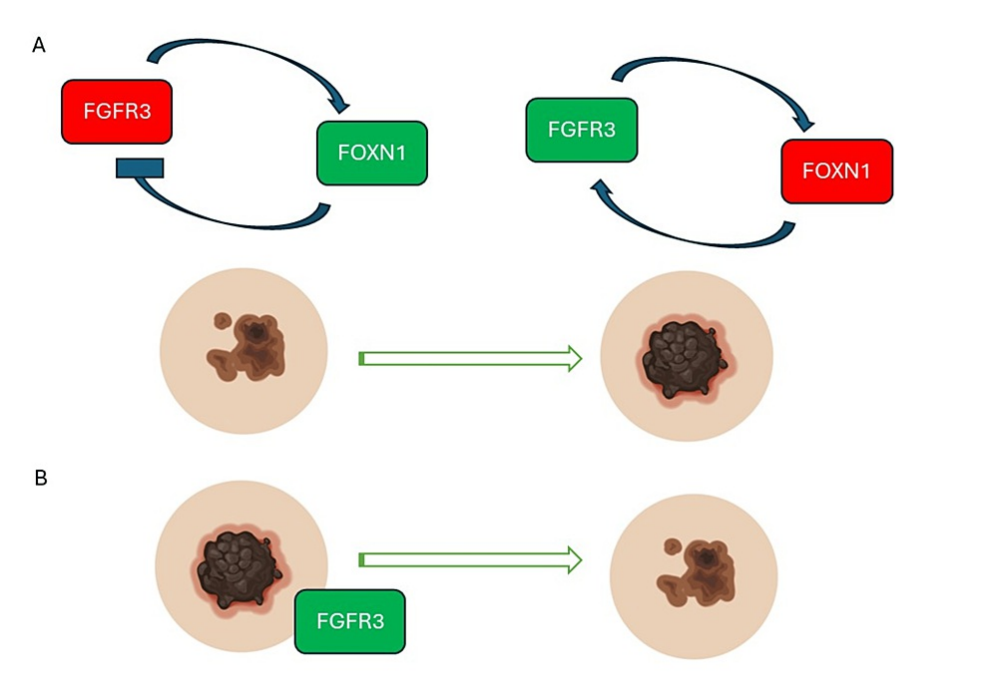
Proposed mechanistic connection between seborrheic keratosis and melanoma. Panel A: The first model suggests instances of melanoma development within SK. The potential mechanistic link involves *FGFR3*, a factor associated with promoting melanoma growth and the most frequently altered gene in both tumors. However, SK shows an additional elevation in the transcription factor *FOXN1*, hinting at a positive feedback loop that may inhibit malignant progression. If a loss-of-function mutation in *FOXN1* or mutations on the promoter occurs, melanoma may develop. Panel B: The second model suggests that a fraction of melanomas may harbor *FGFR3* augmented expression and could potentially acquire SK-like characteristics. Image created using BioRender.

Furthermore, reports indicate instances of melanoma developing within SK [3]. This prompts us to consider a potential mechanistic connection between SK and melanoma. Notably, *FGFR3*, a factor associated with promoting melanoma growth, [6] is also the most frequently altered gene in SK. Despite the common involvement of *FGFR3* in both tumors, SK exhibits an additional elevation in the transcription factor *FOXN1*. This intriguing observation suggests the presence of a positive feedback loop that could potentially inhibit malignant progression [7]. Consequently, we propose that the emergence of a loss-of-function mutation in *FOXN1* or mutations on the promoter might contribute to the transformation of SK into melanoma. Additionally, it is plausible that melanomas harboring *FGFR3* alterations could acquire SK-like characteristics. However, it is important to highlight that this latter aspect remains unexplored to the best of our knowledge (Figure 4).

## Conclusions

Accurate diagnosis of SK and melanoma is crucial to prevent mismanagement. Dermoscopy aids in identifying characteristic features and improving diagnostic precision. The potential interplay between *FGFR3* and *FOXN1* warrants further exploration. Prompt biopsy in uncertain cases is vital for patient safety.

## References

[1] Jackson JM, Alexis A, Berman B, Berson DS, Taylor S, Weiss JS (2015). Current understanding of seborrheic keratosis: prevalence, etiology, clinical presentation, diagnosis, and management. J Drugs Dermatol.

[2] Sun MD, Halpern AC (2022). Advances in the etiology, detection, and clinical management of seborrheic keratoses. Dermatology.

[3] Thomas I, Kihiczak NI, Rothenberg J, Ahmed S, Schwartz RA (2004). Melanoma within the seborrheic keratosis. Dermatol Surg.

[4] Heal CF, Raasch BA, Buettner PG, Weedon D (2008). Accuracy of clinical diagnosis of skin lesions. Br J Dermatol.

[5] Carrera C, Segura S, Aguilera P (2017). Dermoscopic clues for diagnosing melanomas that resemble seborrheic keratosis. JAMA Dermatol.

[6] Li L, Zhang S, Li H, Chou H (2019). FGFR3 promotes the growth and malignancy of melanoma by influencing EMT and the phosphorylation of ERK, AKT, and EGFR. BMC Cancer.

[7] Heidenreich B, Denisova E, Rachakonda S (2017). Genetic alterations in seborrheic keratoses. Oncotarget.

